# Preparticipation Screening of Athletes: The Prevalence of Positive Family History

**DOI:** 10.3390/jcdd10040183

**Published:** 2023-04-21

**Authors:** Bogna Jiravska Godula, Otakar Jiravsky, Petra Pesova, Libor Jelinek, Marketa Sovova, Katarina Moravcova, Jaromir Ozana, Miroslav Hudec, Roman Miklik, Jan Hecko, Libor Sknouril, Eliska Sovova

**Affiliations:** 1 Faculty of Medicine, Palacky University, Krizkovskeho 511/8, 779 00 Olomouc, Czech Republic; 2 Sports Cardiology Center, Nemocnice Agel Trinec-Podlesi, Konska 453, 739 61 Trinec, Czech Republic; 3 Faculty of Medicine, Masaryk University, Kamenice 735/5, 625 00 Brno, Czech Republic; 4 Faculty of Electrical Engineering and Computer Science, VSB-Technical University of Ostrava, 708 33 Ostrava, Czech Republic

**Keywords:** sudden cardiac death, athlete, preparticipation screening

## Abstract

Sudden cardiac death (SCD) is a leading cause of death among athletes, and those with a positive family history (FH) of SCD and/or cardiovascular disease (CVD) may be at increased risk. The primary objective of this study was to assess the prevalence and predictors of positive FH of SCD and CVD in athletes using four widely used preparticipation screening (PPS) systems. The secondary objective was to compare the functionality of the screening systems. In a cohort of 13,876 athletes, 1.28% had a positive FH in at least one PPS system. Multivariate logistic regression analysis identified the maximum heart rate as significantly associated with positive FH (OR = 1.042, 95% CI = 1.027–1.056, *p* < 0.001). The highest prevalence of positive FH was found using the PPE-4 system (1.20%), followed by FIFA, AHA, and IOC systems (1.11%, 0.89%, and 0.71%, respectively). In conclusion, the prevalence of positive FH for SCD and CVD in Czech athletes was found to be 1.28%. Furthermore, positive FH was associated with a higher maximum heart rate at the peak of the exercise test. The findings of this study revealed significant differences in detection rates between PPS protocols, so further research is needed to determine the optimal method of FH collection.

## 1. Introduction

Exercise has many benefits for athletes, including improved brain health, weight management, reduced risk of disease, and stronger bones and muscles [1]. Regular physical activity is recommended by the World Health Organization to improve overall health [2]. However, overexercise can have serious consequences, such as adverse structural and electrical remodeling of the heart, including fibrosis and the stiffening of the atria, right ventricle, and large arteries [3]. Additionally, exercise addiction can harm the body’s tendons, ligaments, immune system, etc. [4]. Athletes, especially active young adults, need to be made aware of these risks when exercising [5].

Sudden cardiac death (SCD) is a leading cause of death in athletes, particularly in young athletes [6,7,8]. The most effective way to reduce the risk of SCD in athletes is through establishing effective resuscitation protocols and increasing the availability of automated external defibrillators [9]. Regular exercise has been shown to significantly reduce the risk of cardiovascular death, including SCD, compared with a sedentary lifestyle [10,11]. Parents and coaches should be familiar with the risk factors and warning signs of an underlying cardiac issue [12].

Preparticipation screening (PPS) is an important tool to reduce the risk of sudden cardiac death in athletes [13,14]. An Italian study found that the incidence of sudden cardiovascular death in young competitive athletes has significantly decreased since the introduction of a nationwide systematic screening [15]. However, the recommendation of PPS for the mass population is still under discussion, primarily due to a lack of evidence of the efficacy and cost-effectiveness of PPS worldwide [16]. In Italy, PPS is mandatory for competitive athletes, and the Italian PPS is known for its comprehensive clinical evaluation, which includes orthopedic assessments, lung function tests, ECG, and urine analysis [17]. This approach has proven successful in reducing the incidence of sudden death in athletes, and its success has influenced the European Society of Cardiology to adopt a common European protocol and the International Olympic Committee to recommend ECG screening for Olympic athletes [17]. Although echocardiographic exams are not mandatory for eligibility, some experts suggest that athletes undergo at least one echocardiographic exam in their lifetime before starting sports activity to further assess the potential risks [18]. However, the Italian PPE has not been universally adopted due to concerns about the high cost/efficacy ratio and the low incidence of fatal acute cardiac events in competitive athletes. Furthermore, the authors suggest that extending the PPE globally to all young people, including non-competitive sport practitioners, would be more reasonable in maintaining the health and safety of the population. Thus, further research is required to establish the benefits of implementing PPS in various regions of the world. Nevertheless, PPS can help raise awareness of cardiac diseases and empower athletes to make informed decisions about their health risks.

The American Heart Association (AHA), Preparticipation Physical Evaluation (PPE-4), International Olympic Committee (IOC), and Fédération Internationale de Football Association (FIFA) are four widely used PPS systems. The AHA recommends testing to assess the risk of SCD and cardiovascular disease (CVD) for athletes and active individuals [19]. Another system is the Preparticipation Physical Evaluation (PPE). The latest edition, the Preparticipation Physical Evaluation (PPE-5) Monograph, was published in 2019 [20] and serves as a resource for medical providers in conducting a comprehensive examination that includes cardiovascular, nervous, musculoskeletal, and other systems. This update supersedes the previous edition, PPE-4, published by the American College of Sports Medicine in 2010. The International Olympic Committee (IOC) [21] and Fédération Internationale de Football Association (FIFA) [22] also have their own preparticipation screening systems to ensure athletes’ safety and health during their participation in sports.

An athlete with a family history of SCD and CVD may be at an increased risk. The guidelines for screening athletes recommend considering family history and other risk factors when assessing eligibility for sports. An appropriate approach to obtaining relevant data is required when collecting family history [23].

Preparticipation screening systems typically investigate a person’s family history of SCD and premature CVD through a self-reported questionnaire administered by a physician. All four PPS systems (PPE, AHA, IOC, and FIFA) include a self-reported family history questionnaire as part of their preparticipation screening protocol. However, there may be differences in the specific questions asked and the level of detail requested about relatives and their medical history.

This study aims to explore similarities and differences in how preparticipation screening systems assess an athlete’s family history of SCD and CVD. The primary objective was to assess the prevalence and predictors of positive FH of SCD and CVD in Czech athletes using four widely used preparticipation screening (PPS) systems. The secondary objective was to mutually compare the functionality of the screening systems.

## 2. Methods

### 2.1. Athlete Cohort

The analysis included all consecutive athletes who underwent preparticipation screening between January 2015 and June 2022 at two sports medicine outpatient clinics in Olomouc and Trinec, Czech Republic. There was no selection according to sex, and the study population reflects the athlete population in the Czech Republic, which is predominantly male. Some athletes underwent multiple screenings during the study period. Athletes who were negative for family history in one year were not excluded from future years, as they may have developed a positive family history for natural reasons, such as aging or newly diagnosed family members with SCD/CVD. Once a positive family history was identified, the subject’s subsequent screenings were excluded from the study.

After applying the exclusion criteria, a total of 13,876 athletes were included in the study, 10,108 (72.8%) of whom were male, and 3768 (27.2%) were female.

Each participant screening consisted of the collection of demographic and clinical data, including gender, age, weight, height, and body mass index (BMI); family and medical history questionnaires; and a bicycle ergometer exercise test with a ramp protocol. In the exercise test protocol, athletes were instructed to perform at their subjective maximum effort. They were encouraged to achieve their maximal effort during the test, and the maximal power in watts attained by each athlete was shared with their coaches. This approach ensured that all athletes were striving to reach their maximal effort, providing a more homogenous population in terms of their training level. Additionally, we calculated the percentage of athletes who achieved at least 85% of their predicted maximal heart rate using the formula ((220 − age) × 0.85) as a surrogate marker for maximum effort. This was carried out to address concerns regarding the athletes’ effort levels during the exercise test. The percentages of athletes reaching this threshold were calculated for the total cohort as well as for the FH+ and FH- subgroups. A univariate analysis using the chi-squared test was conducted to compare the results between the two subgroups.

The blood pressure and heart rate were measured at rest and during peak exercise. Information on each participant’s main type of sport was also collected and classified into four categories according to the 2020 European Society of Cardiology guidelines [24]: mixed, power, endurance, and skill. Data were collected retrospectively from electronic medical records and databases.

### 2.2. Family History Evaluation

The family histories of athletes were evaluated using the four well-known questionnaires mentioned above, which were adopted from the preparticipation systems of the International Olympic Committee (IOC), American Heart Association (AHA), and Fédération Internationale de Football Association (FIFA), as well as the PPE-4 questionnaire.

Each of these systems has specific age limits for defining a positive family history of SCD and CVD occurrence: AHA, IOC, and PPE-4 systems define a positive family history as having a CVD or SCD occurrence at or below the age of 50; the PPE-5 system has a more stringent age limit of 35; and the FIFA system differentiates between genders, defining positive family history for males as under 55 years and for females as under 65 years.

Although the PPE-5 was published during the study period, the PPE-4 was used for all evaluations to maintain the consistency, uniformity, and homogeneity of the data. Athletes with a positive family history in any of these systems were classified as FH+, while others were defined as FH-. For exploratory purposes, a retrospective analysis of medical records was performed to manually extract family history information according to the PPE-5 system.

### 2.3. Statistical Analysis

Statistical analysis was performed using IBM SPSS Statistics for Windows, version 28.0.0.0 (Armonk, NY, USA: IBM Corp). Descriptive statistics were used to summarize the demographic and clinical characteristics of the athlete cohort, including the median and interquartile range for continuous variables and numbers and percentages for categorical variables. Differences between FH+ and FH- athletes were assessed using appropriate non-parametric tests, such as the Mann–Whitney U test for continuous variables and the chi-squared test for categorical variables. A *p*-value < 0.05 was considered statistically significant.

Univariate logistic regression was used to identify any significant associations between individual demographic and health factors and a positive family history of SCD + SVD. Variables that were statistically significant (*p* < 0.05) were then included in a multivariate logistic regression model, with positive family history as the dependent variable and the significant variables as independent variables. Odds ratios (ORs) and 95% confidence intervals (CIs) were calculated to estimate the associations between the independent variables and a positive family history of SCD + CVD.

The proportion of athletes with a positive family history of SCD + CVD was expressed in absolute numbers and percentages for each preparticipation screening system, and for a combination of two, three, or all four systems to allow for comparisons across the different systems. Pairwise comparisons with Bonferroni correction were used to determine whether there were significant differences in the proportion of athletes with a positive family history across the different PPS systems.

## 3. Results

A total of 13,876 athletes underwent preparticipation screening during the study period. The majority of the athletes were male (73%), aged 14 years (IQR 7 years), and participated in mixed sports (86%). Other demographic and clinical variables, as well as exercise test results, are presented in Table 1 (left side). The athletes in our cohort were in good physical condition, reaching the median power-to-weight ratio of 4 W/kg.

A positive family history according to at least one of the PPS systems was identified in 177 (1.28%) of the 13,876 athletes screened. The comparison results of FH+ and FH- athletes are shown in Table 1 (right side). Significant differences were observed in gender distribution, sport type, and maximum heart rate (all *p* < 0.001) between the two groups. As the FH+ group had a higher proportion of male athletes than the FH- group (87% vs. 73%), the mean BMI, weight, and height were also statistically higher in the FH+ group. Other demographic, resting, and exercise characteristics were not significantly different between the two groups.

We further investigated the differences in weight, height, and BMI between the FH+ and FH- groups, stratified by gender. The results of the non-parametric Mann–Whitney U test are presented in Table 2.

The analysis revealed significant differences in weight for females, and in BMI for males between the FH+ and FH- groups. No significant differences were observed in height for either gender. These results suggest that the observed differences in weight, height, and BMI between the FH+ and FH- groups might be partially attributed to the higher proportion of males in the FH+ group.

Multivariate logistic regression analysis showed that the maximum exercise heart rate was a statistically significant predictor of a positive family history of SCD + CVD (OR = 1.042, 95% CI = 1.027–1.056, *p* < 0.001). Height was marginally significant (OR = 0.948, 95% CI = 0.899–0.999, *p* = 0.045), but its effect size was small. No other statistically significant variable was found (Table 3).

**Figure 1:**
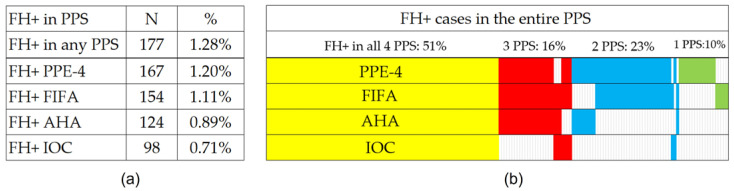
Prevalence of positive family history in the PPS systems: (**a**) this figure shows the overall prevalence (in numbers and percentages) of positive family history in each of the four PPS systems in separate rows (PPE-4, FIFA, AHA, IOC); (**b**) this figure shows the combined prevalence rate in all four systems, three systems, two systems, and one system. Rectangles of different colors represent the distribution of cases with a positive family history in each PPS system.

Regarding the PPS systems and their FH detection functionality, the PPE-4 protocol was the one with the highest number of FH+ cases detected (167 athletes; 1.20%), followed by FIFA (154; 1.11%), AHA (124; 0.89%), and IOC (98; 0.71%) protocols. The exact distribution of positive results among the PPS systems for all 177 FA+ athletes is graphically shown in Figure 1. Pairwise comparisons revealed statistically significant differences in the results between the PPS systems (significance adjusted for multiple testing; resulting in all PPS pairs at *p* < 0.001; the IOC-AHA pair at *p* = 0.002), except for the PPE-4 and FIFA pair (*p* = 0.42); (Supplementary Table S1).

## 4. Discussion

Family history screening is an essential component of preparticipation screening in athletes, as it can identify those at increased risk of SCD and help guide further evaluation and management. While published data suggest up to a two-fold increased risk of SCD in individuals with a family history of SCD, similar data, to the best of our knowledge, have not been published in the athlete population [25,26,27]. The aim of our study was to evaluate the prevalence of a positive family history of SCD and premature CVD in young athletes and to compare the efficacy of four PPS systems. We found a very low prevalence of a positive family history of SCD and premature CVD, suggesting that subsequent cardiac follow-up of FH+ athletes should not challenge the capacity of, or limit access to, healthcare systems. Furthermore, the results highlight the need for ongoing research to explore the relationship between positive FH and the risk of SCD and premature CVD in athletes.

After analyzing four different PPS systems, our study found significant differences in the positive rates of family history for SCD and premature CVD among young athletes. Specifically, just over half (51%) of the athletes were flagged for positive FH across all four PPSs, while 16%, 23%, and 10% were identified by three, two, and one PPS, respectively. These results suggest that assessing an individual’s family history can be challenging, with a high likelihood of misidentifying an individual depending on the PPS selected. In this study, we chose to identify the maximum number of FH+ athletes by utilizing a combination of all four PPSs and establishing the family history status based on a positive result in at least one of them. This approach was used to determine the feasibility of future research, which is highly dependent on available healthcare resources. The results highlight the need to develop a more reliable and evidence-based questionnaire to identify athletes at risk for SCD and premature CVD while minimizing the number of athletes referred for unnecessary cardiac evaluation.

An important consideration in our study is the possibility that the detection of a positive family history in a specific type of screening, not identified by the previous one, could be attributable to a new SCD/CVD event in the athlete’s family or the failure of the previously adopted screening. This highlights the importance of the continuous monitoring and updating of family history information during the preparticipation screening process, as new information about family members’ health conditions can have a significant impact on an athlete’s risk assessment. Our results underscore the need for a comprehensive and regularly updated family history assessment to ensure the accurate identification of athletes with a positive family history of SCD/CVD.

In evaluating a potential association between FH status and exercise test results, the study showed that the maximum heart rate was a statistically significant predictor of a positive FH of SCD + SVD, with a one-beat-per-minute increase in the maximum heart rate at the peak of the exercise test associated with a 4.2% increase in the odds of positive FH. While the maximum heart rate varies innately among individuals, it is not known whether a higher maximum exercise heart rate is associated with an increased risk of SCD in athletes with a positive FH [28]. Therefore, further studies are needed to investigate the relationship between the maximum heart rate and the risk of SCD + SVD in athletes with a positive family history.

Univariate analysis showed that weight, height, and BMI significantly differed between the FH+ and FH- groups. A possible explanation could be the significantly higher proportion of males in the FH+ group, resulting in larger average individual body dimensions. However, in multivariate analysis, only height was marginally significant (OR = 0.948, 95% CI = 0.899–0.999, *p* = 0.045), with a small effect size, and probably without clinical applicability. It is interesting to compare our results with published findings indicating that a higher BMI in adolescence is associated with an increased risk of cardiomyopathy in adulthood [29]. Our observation of a higher representation of athletes with a higher BMI in the FH+ athlete cohort is surprising and raises the question of whether athletes with a known family history undergo less rigorous training and lifestyle, resulting in a higher BMI. However, the direction of causality is unclear, and further research is needed to investigate the potential relationship between BMI, positive family history, and cardiovascular risk in athletes.

Furthermore, the fact that only 27.2% of our cohort were women highlights a concerning disparity in preparticipation screening for female athletes. This gap between the sexes may be due to a number of factors, such as lower participation rates, less emphasis on women’s sports, and social and cultural biases that prioritize male athletes. However, it is important to recognize that sex-specific diseases and phenotypes can play a crucial role in the development of genotype-associated diseases, making preparticipation screening especially important for female athletes.

Despite the fact that the published average annual incidence of sport-related sudden cardiac arrest in women was 0.19 per million, which is more than 10 times lower than in men (2.63 per million) [28], our study confirms that female athletes have a similar risk of a positive family history of SCD and CVD as their male counterparts. Therefore, increased efforts in sports medicine are necessary to ensure equal access to preparticipation screening and care for all athletes. This includes developing evidence-based questionnaires and protocols that are specifically tailored to the needs of female athletes, as well as addressing any social and cultural barriers that may limit their access to screening. Improving preparticipation screening for female athletes is not only important for their own health and well-being but also for the health of future generations of athletes. By promoting equal access to screening and care for all athletes, we can ensure that the benefits of participation in sports are available to everyone, regardless of sex or gender.

In 2019, the American College of Sports Medicine and other medical and professional societies published the fifth version of the Preparticipation Physical Examination (PPE-5) questionnaire [20], which replaced the previous version, PPE-4, that we used in our study. Nevertheless, after this update, we aimed to retrospectively evaluate, where possible, the family history of SCD and CVD using the PPE-5, if possible. The prevalence of FH+ athletes ranged from 0.14% to 0.25% and was the lowest among all the PPS systems, as presented in Supplementary Figure S1. However, it is important to note that our retrospective analysis had some uncertainty, and in some cases, it was not possible to accurately determine whether the family history was positive or normal. The PPE-5 is the most recent and specific questionnaire available, mainly due to the suppression of the age limit for sudden cardiac death in first-degree relatives under 35 years of age. Further research is needed to investigate the sensitivity and specificity of the PPE-5 in a long-term follow-up.

Our study provides valuable insights into the prevalence of family history as a risk factor for sudden cardiac death and cardiovascular disease in young athletes. Our findings highlight the importance of family history screening as an essential component of preparticipation screening in athletes, as it can identify those at increased risk of SCD and help guide further evaluation and management. The low prevalence of a positive family history detected in this study using a combination of four PPS systems or one system alone demonstrates that the subsequent low number of indicated cardiac follow-ups should not challenge the capacity of the healthcare system or limit its availability. However, it also highlights the need for a more reliable, evidence-based questionnaire for identifying athletes with a positive family history of SCD and premature CVD. The use of advanced analytical methods such as big data analysis, machine learning, or deep neural networks could be used to identify even subtle factors associated with SCD in athletes.

Although we focused on evaluating the prevalence of a positive family history of SCD and premature CVD and comparing the efficacy of four PPS systems, we recognize the potential benefits of exploring improved screening criteria and incorporating genetic data. Incorporating genetic data through genome-wide association studies (GWAS) or exon sequencing could potentially enhance the accuracy and reliability of identifying athletes at risk. However, such analyses are beyond the scope of our current study. We recommend that future research should investigate the integration of genetic data into screening protocols, which could potentially lead to more accurate identification of at-risk athletes and guide personalized management strategies.

Moreover, we agree that refining the criteria for positive family history screening is necessary, and we encourage future research to focus on identifying and validating new markers or criteria that can be used in screening protocols. This could involve the use of advanced analytical methods such as big data analysis, machine learning, or deep neural networks to identify even subtle factors associated with SCD in athletes. We believe that such efforts could contribute to the development of more reliable and evidence-based questionnaires for identifying athletes with a positive family history of SCD and premature CVD.

Additionally, long-term follow-up of athletes with a positive family history could provide valuable insights into the role of positive family history in the development of SCD and premature CVD. Tracking the health outcomes of these athletes over time could help to determine the most effective management strategies and further refine screening protocols to ensure the identification of at-risk individuals while minimizing unnecessary evaluations and interventions.

Our study also highlights the need for ongoing research to explore the relationship between athletes’ positive family history and the risk of SCD and premature CVD, as well as to investigate the potential relationship between athletes’ BMI, positive family history, and cardiovascular risk. Additionally, addressing the issue of the under-representation of women in preparticipation screening for athletes is crucial to ensure equal access to preparticipation screening and care for all athletes.

In light of these findings, we recommend that further research should focus on the development of more effective risk assessment tools and further studies in different populations and settings to establish the generalizability of these findings. Ultimately, the goal of this research is to improve preparticipation screening protocols and reduce the risk of SCD and premature CVD in young athletes.

## 5. Conclusions

In a large cohort of young athletes undergoing preparticipation screening, the prevalence of a positive family history of SCD and CVD was low (1.28%). We found a significant association between a higher maximum exercise heart rate and a positive family history. The study revealed inconsistencies among the four acknowledged preparticipation screening protocols, highlighting the need for a more reliable, evidence-based protocol. Future research is needed to fill the gaps in the screening of female athletes and to explore the relationship between positive family history, preparticipation screening results, and risk of SCD and premature CVD based on long-term follow-up of these athletes.

## 6. Limitations

This study had several limitations: (1) The sample population consisted of active athletes from two sports medicine clinics in the Czech Republic who volunteered or were referred for screening for various reasons, resulting in a potential bias between real and found positive family history prevalence. However, we consider this bias negligible and the results representative due to the nature of the healthcare system and screening requirements in the region; (2) although the sample size was large, the generalizability of the results to non-athletes and other populations in different countries may be limited; (3) the relatively low representation of female athletes (27%) in the study cohort may limit the generalizability of the findings to female athletes; (4) the retrospective nature of data collection may have introduced recall bias and data collection errors; (5) the study used the PPE-4 questionnaire and not the latest version, PPE-5, which was published during the enrollment period. Nevertheless, the authors presented an exploratory analysis using PPE-5; and (6) caution should be exercised in interpreting the statistical analysis, as some *p*-values were near the significance threshold, and multiple comparisons were performed.

## Figures and Tables

**Table 1:** Demographics of the entire athlete cohort and stratification by SCD/CVD family history status. The table includes data on sex (female or male), sport type (mixed, skill, endurance, and power), age, weight, height, body mass index (BMI), blood pressure at rest (systolic (BPsystol_rest) and diastolic (BPdiastol_rest)), heart rate at rest (HR_rest), maximum heart rate at the peak of the exercise test (HR_max), maximum workload (in watts per kilogram of body weight), and systolic blood pressure workload slope (SBP_W_slope, in mmHg/(W/kg)).

		Total		FH Positive		FH Negative		*p*
		N	%	N	%	N	%	
Sex (N; %)	Female	3768	27.2%	23	13%	3745	27%	<0.001
	Male	10,108	72.8%	154	87%	9954	73%	
Sport type (N; %)	Mixed	3084	86.2%	135	76%	2949	87%	<0.001
	Skill	18	0.5%	8	5%	10	0%	
	Endurance	299	8.4%	25	14%	274	8%	
	Power	175	4.9%	9	5%	166	5%	
Achievement of 85% predicted maximal heart rate (N; %)		11,230	87.5%	156	92%	11,074	87%	0.113
		median	IQR	median	IQR	median	IQR	
Age on the exam date (years)		14	7	14	5	14	7	0.876
Weight (kg)		57.9	30.8	63.4	26	57.8	30.9	0.007
Height (cm)		166	25	170	21	166	24.7	0.005
BMI (kg/cm^2^)		20.5	5.9	21.6	5.5	20.5	5.9	0.011
BPsystol_rest (mmHg)		120	20	120	15	120	20	0.093
BPdiastol_rest (mmHg)		70	15	70	10	70	15	0.878
HR_rest (/min)		78	19	78	31	78	19	0.451
HR_max (/min)		186	17	190	12	186	17	<0.001
BPsystol_max (mmHg)		160	35	160	22	160	35	0.142
maximal workload (W/kg)		4.0	1.0	4	0.5	4	1	0.729
SBP_W_slope (mmHg/(W × kg))		0.18	0.11	0.20	0.08	0.18	0.11	0.151

**Table 2:** Differences in weight, height, and BMI between the FH+ and FH- groups, stratified by gender. The table presents the mean and standard deviation (SD) of weight (in kg), height (in cm), and body mass index (BMI, in kg/cm^2^) for the total study population, the FH+ group, and the FH- group, separated by gender. The *p*-values in the rightmost column indicate the statistical significance of the differences between the FH+ and FH- groups, assessed using the non-parametric Mann–Whitney U test.

		Total		FH Positive		FH Negative		Stats
		Mean	SD	Mean	SD	Mean	SD	*p*=
Weight (kg)	Female	56.8	17.4	65.1	20.5	56.8	17.3	0.042
	Male	59.0	21.6	61.5	18.4	58.9	21.7	0.065
Height (cm)	Female	161	13	168	17	161	12	0.075
	Male	165	18	167	14	165	18	0.166
BMI (kg/cm^2^)	Female	21.4	4.9	22.5	4.2	21.4	4.9	0.14
	Male	20.9	4.5	21.5	3.8	20.9	4.5	0.022

**Table 3:** Multivariate logistic regression analysis of factors associated with a positive family history of sudden cardiac death and cardiovascular disease (SCD + SVD) in athletes. The table shows the results of multivariate logistic regression analysis to identify factors associated with a positive family history of SCD + SVD in a cohort of 13,876 athletes. The analysis includes variables such as sex, sport type, weight, height, body mass index (BMI), and maximum heart rate (HR_max). The table presents the regression coefficient (B), standard error (SE), Wald statistic, degrees of freedom (df), significance level (Sig.), and odds ratio (Exp (B)) with corresponding 95% confidence intervals (CIs). A *p*-value < 0.05 was considered statistically significant.

	B	SE	Wald	df	Sig.	Exp (B)	Lower 95% CI for Exp (B)	Upper 95% CI for Exp (B)
Sex	−0.102	0.262	0.151	1	0.698	0.903	0.540	1.511
Sport type	−0.142	0.087	2.697	1	0.101	0.867	0.732	1.028
Weight	0.066	0.039	2.954	1	0.086	1.069	0.991	1.152
Height	−0.054	0.027	4.030	1	0.045	0.948	0.899	0.999
BMI	−0.200	0.112	3.212	1	0.073	0.819	0.658	1.019
HR_max	0.041	0.007	33.416	1	0.000	1.042	1.027	1.056

## Data Availability

The data presented in this study are available on request from the corresponding author.

## Supplementary Materials

The following supporting information can be downloaded at: https://www.mdpi.com/article/10.3390/jcdd10040183/s1, Figure S1: Prevalence of positive family history in PPS systems including PPE-5: (a) this figure shows the overall prevalence (in numbers and percentages) of positive family history in each of the five PPS systems (PPE-5, PPE-4, FIFA, AHA, and IOC) in separate rows; (b) rectangles of different colors represent the distribution of cases with positive family history in each PPS system. The purple color in PPE-5 indicates probable positive family history and the grey color indicates possible positive family history using PPE-5. Note the uncertainty in the PPE-5 results due to retrospective analysis; Table S1: Statistical comparison of positive family history analysis between four different PPS systems. The results of the pairwise comparison between the four PPS systems (PPE-4, FIFA, AHA, and IOC) in terms of significance and adjusted significance (via Bonferroni correction for multiple testing).
