# Supplementary material for: Preparticipation Screening of Athletes: The Prevalence of Positive Family History
Source: J Cardiovasc Dev Dis. 2023 Apr 21;10(4):183. doi: 10.3390/jcdd10040183 (PMC10144243; doi:10.3390/jcdd10040183)
Supplement: Supplementary file 1 [file jcdd-10-00183-s001.zip › jcdd-2320893-supplementary.pdf]

Supplementary Materials:

Sample 1-Sample 2	Test Statistic	Std. Error	Std. Test Statistic	Sig.	Adj. Sig.[a]
IOC-AHA	0.147	0.040	3.647	0.000	0.002
IOC-FIFA	−0.316	0.040	−7.854	0.000	0.000
IOC-PPE-4	0.390	0.040	9.678	0.000	0.000
AHA-FIFA	−0.169	0.040	−4.208	0.000	0.000
AHA-PPE-4	−0.243	0.040	−6.031	0.000	0.000
FIFA-PPE-4	0.073	0.040	1.823	0.068	0.410

Each row tests the null hypothesis that the Sample 1 and Sample 2 distributions are the same. Asymptotic significances (2-sided tests) are displayed. The significance level is .050.

[a] Significance values have been adjusted by the Bonferroni correction for multiple tests.

Supplementary Table S1: Statistical comparison of positive family history analysis between four different PPS systems. The results of the pairwise comparison between the four PPS systems (PPE-4, FIFA, AHA, IOC) in terms of significance and adjusted significance (by Bonferroni correction for multiple testing).

FH+ in PPS	N	%
FH+ in any PPS	177	1.28%
FH+PPE-5	19-34	0.14-0.25%
FH+ PPE-4	167	1.20%
FH+ FIFA	154	1.11%
FH+ AHA	124	0.89%
FH+ IOC	98	0.71%

(a)

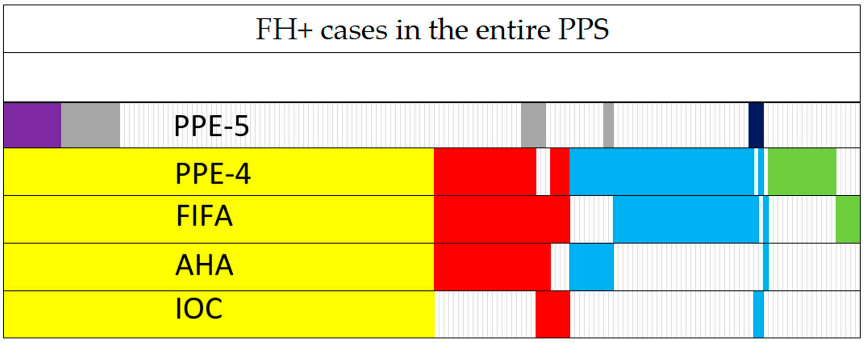

(b)

Supplementary Figure S1: Prevalence of positive family history in PPS systems including PPE-5. (a) The figure shows the overall prevalence (in numbers and percentages) of positive family history in each of the five PPS systems (PPE-5, PPE-4, FIFA, AHA, IOC) in separate rows. (b) Rectangles of different colors represent the distribution of cases with positive family history in each PPS system. The purple color in PPE-5 indicates probable positive family history and the grey color indicates possible positive family history using PPE-5. Please note the uncertainty in the PPE-5 results due to retrospective analysis.
